# Comparison of Internal Accuracy of Ceramic Veneered Sintered Metal versus Cast Metal Restoration Upon Different Fabrication Processes

**DOI:** 10.1055/s-0045-1807730

**Published:** 2025-05-01

**Authors:** Sanephume Sripairojn, Niwut Juntavee, Apa Juntavee

**Affiliations:** 1 Division of Biomaterials and Prosthodontics Research, Faculty of Dentistry, Khon Kaen University, Khon Kaen, Thailand; 2 Department of Prosthodontics, Faculty of Dentistry, Khon Kaen University, Khon Kaen, Thailand; 3 Division of Pediatric Dentistry, Department of Preventive Dentistry, Faculty of Dentistry, Khon Kaen University, Khon Kaen, Thailand

**Keywords:** ceramometal, digital impression, internal accuracy, press-on ceramic, sintered metal

## Abstract

**Objectives:**

One critical factor that influences clinical outcomes of fixed dental restorations is the internal gaps between the restoration and the abutment tooth. However, investigating these gaps in the context of fabrication processes with new technologies is few. This study compared internal accuracy of sintered versus cast metal substructures upon different fabrication techniques, veneered with layering and press-on ceramic, during different construction stages, at different sites of restoration.

**Materials and Methods:**

A total of 96 metal substructures were fabricated with a standardized dimension from four techniques: cast metal with traditionally impressed tooth [CmTt], cast metal with digitally milled wax [CmDw], sintered metal with digitally impressed tooth [SmDt], and sintered metal with digitally impressed stone model [SmDm]. They were further subdivided into two subgroups according to the veneering ceramic used [layered (Pl) and press-on (Pp)]. Internal accuracy was evaluated at gingival, gingiva-axial, axial, axio-occlusal, and occlusal locations using silicone replica, after metal coping (As), degassing (De), opaque application (Op), contouring (Co), and glazing (Gl).

**Statistical Analysis:**

Analysis of variance and Bonferroni tests were analyzed for significant differences of internal fit upon different factors ( *α* = 0.05).

**Results:**

Significantly different internal accuracy was found upon metal substructures fabrication technique, veneering methods, stages, and sites of restoration ( *p* < 0.05). SmDt and SmDm revealed significantly better fit than CmTt and CmDw ( *p* < 0.05). Pp generated significantly better fit than Pl ( *p* < 0.05). Significantly increasing gaps were found upon stages ( *p* < 0.05). Occlusal and axio-occlusal sites exhibited larger gaps than axial, gingivo-axial, and gingival sites ( *p* < 0.05). However, all groups exhibited clinically acceptable internal accuracy.

**Conclusion:**

Increasing internal inaccuracies upon stages of fabrication were noticed, with highly observed at the occlusal and axio-occlusal sites. Sintered metal (SmDt, SmDw) provided better accuracy than cast metal (CmTt, CmDw) while press-on veneering generated better accuracy than the layering method. Ceramic press-on sintered metal was suggested for fabrication restoration.

## Introduction

Ceramometal restorations have been successfully utilized for restoring damaged teeth owing to the aesthetic ceramic veneering on a durable metal substructure. 1 Construction of ceramometal restoration comprises of two steps; metal substructure fabrication and ceramic veneering. Predominately, nonnoble alloys are frequently used due to their superior mechanical properties, biocompatibility, and lower cost compared with others. The cobalt-chromium alloys are selected for patient sensitive to nickel. 1 2 Nonnoble metal substructures are conventionally fabricated through the lost-wax technique, which is a considerably sensitive technique and inferior castability, more than noble metals, owing to their high melting temperature and little ductility. 3 Thus, some accumulating errors from the casting processes are unavoidable. Lately, computer-aided design and computer-aided manufacturing (CAD-CAM) technology has allowed for the fabrication of metal substructures through a milling process by using data designed with CAD software. 4 5 6 The systems are capable of producing better accuracy and reliability of restorations from milling presintered alloy blank, however, it requires producing at a qualified milling center, which is costly. 7 Milling the partially sintered powder alloy offers a simpler process but needs further sintering to achieve the fully sintered alloy, which consumes less time and cost. The mechanical properties of the sintered alloy were proved to be comparable to the hard-milled alloy. 8 9 Through CAD-CAM it is now possible to fabricate wax pattern to be further invested and cast or pressed for coping. The accuracy of the sintered metal substructure is probably related to the omission of waxing, investing, and casting processes in the conventional casting method. 10 Nevertheless, limitations of laser scanners in the CAD systems to reproduce sharp edges were reported. 8

Veneering the metal substructure with ceramic can be accomplished either by conventional layering or pressed-on methods. To achieve favorable esthetic and clinical results demands a skilled dental technician. On using the layering method, the ceramic slurry, made from mixing ceramic powder with modeling liquid, is stacked and overbuilt to compensate for firing shrinkage. During the process, the metal substructure is subjected to a series of ceramic firing cycles and exposed to a variety of high-temperature treatments relative to each ceramic layer applied. Thus, metal copings may get affected and undergo distortion. 11 The press-on technique enables easier producing ceramometal restorations by pressing the melted ceramic onto the metal substructure. 12 This technique avoids multiple ceramic firings and results in achieving precise anatomical form and occlusion of the restorations to the opposing tooth. Better distribution of crystalline phases in the glass matrix minimizes ceramic firing shrinkage and provides better internal accuracy. 12

Internal accuracy is defined as the distance between the intaglio surface of the restoration and the prepared tooth surface, which is considered an essential part of the long-term success of fixed restoration. It is significantly influenced by the accuracy of the fabrication process. Improper internal fit provokes microbacteria to spread through the gap, which potentially causes dental caries and periodontal disease that precedes restoration failure. 13 Excessive internal gap induces tensile stresses at the intaglio surface of the restoration while deforming the luting cement upon cyclic load triggers, chipping of veneering ceramic and decreases fracture strength. 14 Conversely, insufficient cement space can impede the proper seating of the restoration 15 and potentially cause a larger gap upon cementation, which leads to biological complications. 16 The distortion of the ceramometal restoration probably increased after ceramic veneering, firings, and glazing, which perhaps prevented the internal adaptation of restorations to abutment. 3 17 18 However, dimensional changes mostly occur during the cooling process. 3 19 This is possibly related to the substructure design, fabrication technique, type of alloy, shrinkage of ceramic, and the coefficient of thermal expansion difference between alloy and ceramic. 1 2 3 6 12 20 Some studies reported the porcelain firing cycle effect on internal accuracy, 21 22 while others did not. 12 17 The consistent internal fit facilitates seating and also maintains retention or resistance of restoration. 17 23 24 Up to the present time, there is no consensus on the clinically acceptable internal accuracy for fixed dental restoration. An internal gap of 50 to 100 μm was suggested for conventional types of cement, 25 whereas 200 to 300 μm for adhesive types of cement. The largest internal gap appeared at the occlusal area, 13 with an acceptable range from 100 to 200 μm was stated. 26 Yet, a suitable internal gap is required to promote acceptable fit and ensure clinical reliability. 14

The impact of ceramometal fabrication techniques on internal adaptation is a crucial concern for dentists. 1 11 24 Both the sintered metal substructure and the press-on ceramic veneering method are promising future of restoration. 5 6 11 Nevertheless, there was limited information on the internal accuracy of ceramic veneer sintered alloy related to ceramic veneering methods, as well as limited connection to practicing processes. 11 Hence, this *in vitro* study intended to assess the internal accuracy of ceramometal crowns fabricated from four metal substructure fabrication techniques, veneered with two ceramic veneering methods, and measured at five stages of fabrication, at 18 sites of restoration, with the research design relevant to clinical practice processes. The null hypothesis was that the internal accuracy of the ceramometal crown not being significantly affected by the difference in metal substructure fabrication techniques, ceramic veneering methods, stages of restoration fabrication, sites of restoration, and their interactions. Conventional ceramometal crowns based on traditional fabricated cast metal veneered with conventional porcelain layering served as a control group.

## Materials and Methods

The sample size was estimated by G*power 3.1 software (Heinrich-Heine-Universität, Düsseldorf, Germany) using the values from the previous study, 27 power of test = 0.9, and an *α* error = 0.05 as shown in Equation 1 .



where *Z _α_* = normal standard deviation = 1.96 ( *α* error = 0.05), *Z _β_* = normal standard deviation = 1.28 ( *β* error = 0.1), *µ*_1_ – *µ*_2_ = differences of mean between tested groups = 5, and *s* = standard deviation ( *s*_1_ = 5, *s*_2_ = 2).

The calculated sample size was 12 specimens/group used for this investigation.

## Fabrication Master Model

**Fig. 1:**
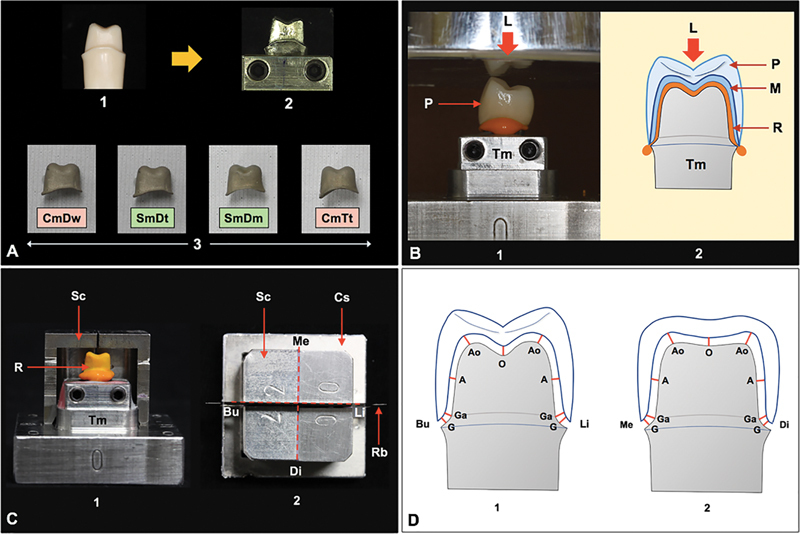
( **A** ) A typodont maxillary first premolar was prepared (1), replicated to a cast metal die, positioned on the metal base model (2), which was used for fabrication of four types of metal substructures including cast metal with traditionally impressed tooth [CmTt], sintered metal with digitally impressed tooth [SmDt], sintered metal with digitally impressed stone model [SmDm], and cast metal with digitally milled wax [CmDw] (3). ( **B** ) The intaglio surface of the ceramic (P) veneer metal (M) crown was filled with a light viscosity silicone impression material, placed onto the master die (Tm), and constantly loaded (L) in apical direction (1, 2). ( **C** ) The silicone replica (R) was picked up with regular viscosity silicone impression material using split mold metal cap (Sc) and cap stabilizer (Cs) (1) for further sectioning in mesial-distal (Me–Di) and buccal-lingual (Bu–Li) directions using razor blades (Rb) (2). ( **D** ) The internal accuracy was determined at gingival (G), gingiva-axial (Ga), axial (A), axio-occlusal (Ao), and occlusal (O) location of the buccal (Bu), lingual (Li), mesial (Me), and distal (Di) sites.

**Fig. 2:**
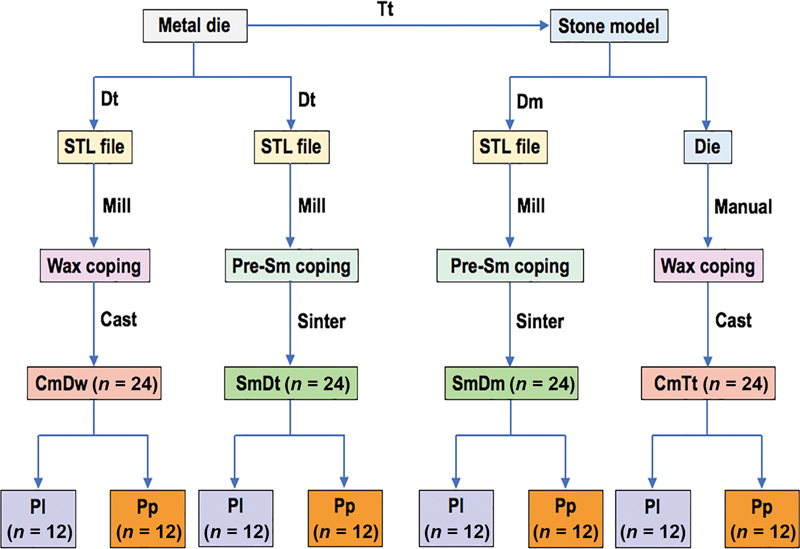
The metal die was traditionally impressed (Tt) to fabricate 48 stone models. Twenty-four standard tessellation language (STL) files were generated from digitally impressed metal tooth (Dt), designed, milled wax (Dw) copings to fabricate cast copings (Cm) upon the CmDw technique. Twenty-four STL files were generated by Dt, designed, milled presintered copings, and sintered to derive sintered metal (Sm) copings upon the SmDt technique. Twenty-four STL files were generated by digitally impressed stone models (Dm), designed, milled presintered copings, and sintered to reach for sintered metal (Sm) copings upon to SmDm technique. Twenty-four stone dies were manually carved for wax copings to fabricate cast metal (Cm) copings upon the CmTt technique. All metal copings were randomly veneered with ceramic by either ceramic layering (Pl) or ceramic press-on (Pp) technique.

A typodont maxillary first premolar (Frasaco, Tettnang, Germany) was prepared for the ceramometal crown with diamond rotary instruments (Khon Kaen University Preparation Kit 1918, Jota, Ruthi, Switzerland) and high-speed handpiece (KaVo, Biberarch, Germany). The preparation was designed for 1.5 mm identical axial and anatomical occlusal reduction, a 1.2-mm smooth continuous chamfer finishing line located 0.5 mm above the cemento-enamel junction with a round internal line angle, and 10 degrees total occlusal convergence angle ( Fig. 1A-1 ). The prepared tooth was duplicated with polyvinyl siloxane (PVS) impression material (Silagum, DMG, Hamburg, Germany) and poured with pattern resin (Duralay, Reliance, Alsip, Illinois, United States). The resin pattern was invested in the casting ring using phosphate bonded investment (Ceramvest Hi-Speed; Protechno, Girona, Spain) to transform to cast metal through the process of loss wax technique. The investment mold was burnt-out in a furnace (EWL-5645, Kavo) and cast with nonnoble metal alloy (d.SIGN 30, Ivoclar Vivadent, Schaan, Liechtenstein) using a centrifuged casting apparatus (Fornax, Bego, Bremen, Germany). The metal casting specimens were divested and sandblasted with 110 μm aluminum oxides (Al _2_ O _3_ ) abrasive (Korox, Bego) in a sandblasting machine (Vario, Renfert, Hilzingen, Germany) to remove the remaining residue. The sprues were cut with Al _2_ O _3_ cutting disk (Shofu, Kyoto, Japan), and the cast was finished with Dura-Green Stone burs (Shofu), polished with gold polishing kit (Shofu), and ultrasonically cleaned with the distilled water for 15 minutes in a cleansing machine (Vitasonic, Vita Zahnfabrik, Bad Sackingen, Germany) to remove the remaining Al _2_ O _3_ particle and cleaned with streaming machine (Touchsteam, Kerr, Brea, California, United States) to eliminate the grease residues. The metal tooth abutment was positioned at the central portion of the metal base (width × length × thick = 3 × 4 × 3 cm) and used as a master model in this investigation ( Fig. 1A-2 ).

## Fabrication of Metal Substructure

Single ceramometal crowns were evaluated for internal accuracy. A total of 96 metal substructures were fabricated with a standard thickness of 0.3 mm with narrow metal collars all around the margin and designated into four main groups: cast metal constructed from the traditionally impressed tooth (CmTt), cast metal constructed from digitally milled wax (CmDw), sintered metal constructed from the digitally impressed tooth (SmDt), and sintered metal constructed from the digitally impressed model (SmDm) ( Fig. 2 ). All substructures were subjected to degassing and opaque application fired according to the manufacturer's instructions. These groups were further subgrouped according to the method of ceramic veneering into conventional layered and press-on. Evaluation of internal accuracy was carried out at different stages of fabrication; after metal substructure construction, after degassing, after opaque application, after veneering, and lastly after glazing, respectively. All laboratory processes for the fabrication of ceramic veneered metal were blindly performed by one well-qualified dental technician who is capable of performing both digital and conventional fixed prosthesis, followed the standardized protocols, and conducted in the professional dental institute under the blind supervision of the Professor of Prosthodontics belonging to the institute. The metal substructure fabrication techniques were described as follows.

### Cast Metal (Cm) Constructed from Traditionally Impressed Tooth (Tt) [CmTt Technique]

Twenty-four traditional impressions of the master die (Tt) were performed by the double-mixed, one-step impression technique using a light viscosity (syringe type) and putty-soft (tray type) PVS impression material (Silagum, DMG) with a customized autopolymerizable resin (Formatray, Kerr) trays and then poured the impression with type IV dental stone (Vel-Mix, Kerr) to fabricate 24 stone models and dies. The hardening solution (Bredent, Senden, Germany) was applied to the stone casts. Two coats of red color die spacer (Durolan, DFS, Riedenburg, Germany) (10 µm thickness per coat application as specified by the manufacturer) were applied on the stone dies with 0.5 mm clearance from the finishing line of the abutments to reach an equivalent thickness of 20 μm and let dry for 60 seconds. Twenty-four wax pattern copings were fabricated with standardized control for identical shape and thickness through a wax-dipping method using the blue inlay casting wax (Kerr). Then, the sculpting wax was added to thin areas by an electric wax carver (SJK 110, Bonew, California, United States), finalized margin with cervical red wax (GEO Crowax, Renfert, Germany), controlled shape of wax pattern coping with jig, verified wax coping of a thickness of 0.3 mm with a wax caliper (Unique Dental Supply, Concord, Ontario, Canada), and transformed to cast metal (Cm) substructures using nonnoble metal alloy (d.SIGN 30, Ivoclar Vivadent, comprising Co 66.2%, Cr 30.1%, Ga 3.9%, Nb 3.2%, Mo 5%, and Si, Fe, Mn < 1%, and Si, B, Fe, Al, Li < 1%) through the loss wax technique as previously described to derive for 24 CmTt copings ( *n* = 24).

### Cast Metal (Cm) Constructed from Digitally Milled Wax (Dw) [CmDw Technique]

Twenty-four digital impressions of the master die were obtained by scanning with a confocal microscopy-based intraoral scanner (TRIOS 5, 3Shape A/S, Copenhagen, Denmark) to produce standard tessellation language (STL) files to be used in designing substructures with the CAD software (Ceramill Mind v2.7.05, Amann Girrbach, Koblach, Austria) by setting marginal discrepancy for 0 mm, thickness of 0.3 mm, and 20 µm simulated die spacer starting 0.5 mm away from the finishing line of the prepared abutment. The intended data were assigned to a 5-axis CAM-milling instrument (Ceramill Motion 2; Amann Girrbach) for milling 24 digitally milled wax (Dw) copings from the hard wax blank (Ceramill Wax, Amann Girrbach) for further fabrication of 24 CmDw copings ( *n* = 24) by means of loss wax technique as previously described.

### Sintered Metal (Sm) Constructed from Digitally Impressed Tooth (Dt) [SmDt Technique]

Twenty-four digital impressions of the master die (Dt) were obtained by scanning with an intraoral scanner (TRIOS 5, 3Shape A/S) to produce STL files, and used for designing substructures with the CAD software (Ceramill Mind v2.7.05, Amann Girrbach), with the previous identical design, but setting 15% larger than required. The proposed data were assigned to mill the presintered Co-Cr alloy blank (Sm, Ceramill Sintron, Amann Girrbach, comprising Co 66%, Cr 28%, Mo 5%, and Si, Fe, Mn < 1%) in a CAM-milling device (Ceramill Motion 2; Amann Girrbach) to produce presintered metal copings and further sintered to derive for 24 SmDt copings ( *n* = 24).

### Sintered Metal (Sm) Constructed from Digitally Impressed Model (Dm) [SmDm Technique]

Twenty-four conventional stone models and dies were fabricated as previously described. The digital impressions of the stone models (Dm) were obtained by scanning with a cast scanning machine (Ceramill Map-400; Amann Girrbach) to produce STL files and used for designing substructures with the CAD software (Ceramill Mind v2.7.05, Amann Girrbach), with the previous identical design, but setting 15% larger than required. The intended data were assigned to a CAM-milling device (Ceramill Motion 2; Amann Girrbach) to produce presintered metal copings from Co-Cr presintered alloy blank (Sm, Ceramill Sintron, Amann Girrbach) and further sintered to derive for 24 SmDm copings ( *n* = 24).

The sintered metal coping for both were further sintered at 1,300°C for 6 hours in an argon gas chamber of the furnace (Ceramill Argotherm-2, Amann Girrbach) for both SmDt and SmDm

## Surface Preparation of Metal Substructure

The surface of metal substructures was prepared by grinding with stone bur (coral stones, Shofu) in a unique path at 20,000 revolutions per minute speed and then blasted with 110 μm Al _2_ O _3_ (Korox, Bego) using 2 bar pressure for 10 seconds in a sandblaster unit (Vario, Renfert) with placing the tip at 10 mm away from the substructure. The samples were ultrasonically cleaned in the distilled water for 30 minutes and then steam-cleaned for 15 seconds. All metal substructures were heat treated in the furnace (Programat, Ivoclar Vivadent) to oxidize the metal surface by degassing process (D) as following the manufacturer's instruction.

## Opaque Ceramic Application

The A3 paste opaque ceramic (IPS Inline, Ivoclar Vivadent) was smeared to all metal copings and fired twice as stated by the company's firing instruction in the ceramic furnace (Programat, Ivoclar Vivadent) to derive for the 0.1-mm thickness of opaque layer. The first opaque ceramic layer was sparsely smeared and fired. The second opaque ceramic layer was completely applied over the first layer and fired to achieve an eggshell appearance surface. Each type of metal substructure was randomly allocated into two subgroups to be veneered with ceramic either by conventional ceramic layering (Pl) or ceramic press-on (Pp) technique ( Fig. 2 ).

## Conventional Ceramic Layering Technique

The samples were veneered with ceramic with Pl technique to the desired shape and thickness. A creamy uniformity of A3 dentine porcelain (IPS InLine, Ivoclar Vivadent) was spread over the opaque surface, condensed with an ultrasonic condensing machine (Ceramosonic, Unitek, Osaka, Japan) to generate crown contour, and fired in the ceramic furnace (Programat, Ivoclar Vivadent) according to the manufacturer's instructions. The dentine ceramic was permitted for two applications to derive the final contour of 1.2 mm thickness, using a jig to control an anatomical crown contour, and finally glazed.

## Ceramic Pressed-On Technique

The samples were coated with the modeling wax (Geo Classic, Renfert) for 1.2 mm thickness to fabricate the anatomical crown contour, attached the sprue to the wax portion, invested in the silicone investing ring, using a phosphate bonded investment (IPS PressVEST Speed; Ivoclar Vivadent), and burned out in the furnace according to the manufacturer's instruction. After the completion of the burned-out process, the investment mold was relocated to a pressed oven (EP 500, Ivoclar Vivadent) for the porcelain pressing (Pp) process, using A3 porcelain ingots (IPS InLine PoM, Ivoclar Vivadent). After the pressed process was accomplished, the divestment process was performed by blasting with 110 µm Al _2_ O _3_ abrasive (Korox, Bego) with a pen blaster (Vario, Renfert) by setting the blasting tip at 20 mm far from the crown surface, with 4 bars pressure, for 10 seconds. Once the pressed ceramic became visible, the pressure was reduced to 1.5 bars, and gently blasted the ceramic surface. A diamond disk (Kerr) was used to separate the sprues from the crown. Then, the samples were finished, polished, and glazed.

## Evaluation Internal Accuracy

All samples were evaluated for internal accuracy on the same master die. The silicone replica method was used to assess the internal accuracy of restoration to the master die (Tm) on the master metal model. 1 28 The internal accuracy was measured by a single operator, based on blind investigation, at each stage of restoration fabrication including as-cast (As) metal coping, degassing (De), opaquing (Op), body contouring (Co), and glazing (Gl). The intaglio surface of the metal coping was inspected under a widefield zoom stereo-microscope (Carl Zeiss, Oberkochen, Germany) to confirm free of residue particles before evaluating the internal gap for every stage. The intaglio surface of the restoration was covered with a light viscosity PVS material (Silagum, DMG), positioned on the Tm, together with constantly loaded for 50 Newton (N) in the vertical direction until the PVS material completely polymerized ( Fig. 1B ). 29 Upon removal of the restoration from the Tm, a skinny layer of silicone replica (R) was left adhering on the Tm, representing the discrepancy between restoration and Tm ( Fig. 1C-1 ). A regular viscosity PVS material (Reprosil, Dentsply Sirona, Charlotte, North Carolina, United States) was applied into the split mold metal cap (C) and seated on the master metal model by keeping in place the cap stabilizer (Cs) to pick up the replica and further injected a regular viscosity PVS material (Reprosil, Dentsply Sirona) to the internal surface to stabilize the R as a sandwiching method. The R was longitudinally sectioned by super thin razor blades (Gillette, Boston, Massachusetts, United States) through the center of the replica in buccal (Bu)–lingual (Li) and mesial (Me)–distal (Di) directions ( Fig. 1C-2 ), leading to four sections of silicone replica used for measuring the internal gap at gingival (G), gingiva-axial (Ga), axial (A), axio-occlusal (Ao), and occlusal (O) location of the buccal (Bu), lingual (Li), mesial (Me), and distal (Di) sites, yielding 18 measurements for each stage by using the polarized light microscope (Eclipse LV100pol, Nikon, Melville, New York, United States) at ×30 magnification. Each measurement was repeated three times, and performed by a single examiner with 89% intraexaminer agreement. The thickness of the silicone replica between the metal coping and the internal surface of the tooth abutment was measured, analyzed by Image J software (U.S. National Institutes of Health, Bethesda, Maryland, United States), and was defined as the internal accuracy ( Fig. 1D ). 2

### Statistical Analysis

The internal gap data were analyzed with statistics software (SPSS/PC V-26, IBM, Armonk, New York, United States). The data were examined for normality using the Shapiro–Wilk test and for the homogeneity of the variances using the Levene's test. Since the data met the assumptions for an analysis of variance (ANOVA), a comparison of the measured adjustments was analyzed using multifactorial ANOVA to conclude the statistically significant difference in internal accuracy of the ceramometal restorations upon different substructure fabrication techniques, ceramic veneering methods, stages of fabrication, and sites of restoration. Post hoc Bonferroni multiple comparisons were applied to justify differences among groups at a 95% level of confidence.

## Results

**Fig. 3:**
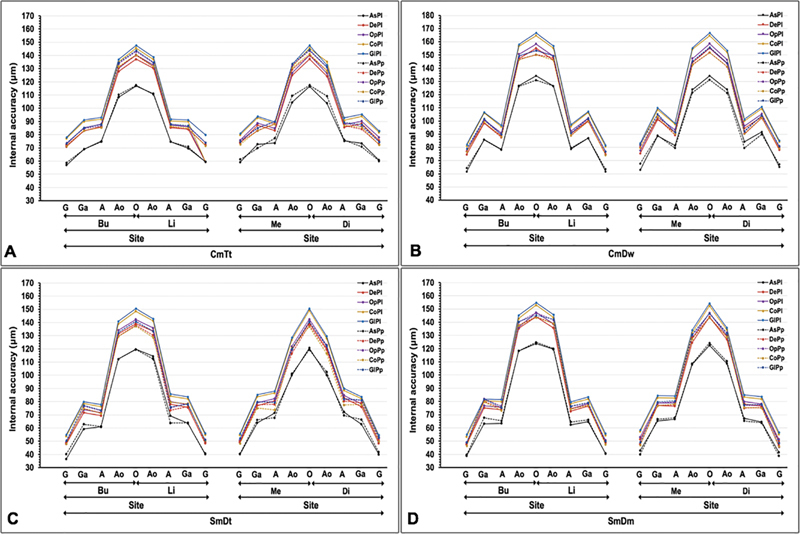
Mean internal accuracy at different sites [gingival (G), gingiva-axial (Ga), axial ( **A** ), axio-occlusal (Ao), and occlusal (O) location of the buccal (Bu), lingual (Li), mesial (Me), and distal (Di) surface] of ceramic veneered ( **A** ) cast metal constructed from traditional loss wax (CmTt), ( **B** ) cast metal of the digitally impressed wax (CmDw), ( **C** ) sintered metal of digitally impressed tooth (SmDt), and ( **D** ) sintered metal of digitally impressed model (SmDm) techniques, with either ceramic layering (Pl) or ceramic press-on (Pp) at as-cast (As), degassing (De), opaquing (Op), body contouring (Co), and glazing (Gl) stages.

The means internal accuracy for ceramometal restorations related to different metal substructure fabrication techniques, stages of restoration fabrication, methods of ceramic veneering, and sites of restoration were presented ( Table 1 and Fig. 3 ). Multifactorial ANOVA confirmed significantly different internal accuracy of restoration upon various substructure fabrication techniques, stages of restorative fabrication, methods of ceramic veneering, and sites of restoration ( *p* < 0.05). When interacting factors were taken into account, significant differences were detected between the substructure fabrication techniques and the methods of ceramic veneering, between the substructure fabrication techniques and sites of restoration, between the substructure fabrication techniques and stages of restoration fabrication, between the methods of ceramic veneering and the sites of restoration, between the methods of ceramic veneering and the stages of fabrication, together with between the substructure fabrication techniques, methods of ceramic veneering, and the sites of restoration ( *p* < 0.05). However, no significant differences in internal accuracy were detected between the sites of restoration and the stages of fabrication, and among other three and four factors interaction ( *p* > 0.05) ( Table 2 ).

**Table 1:** Mean internal accuracy (μm) of cast (Cm) and sintered (Sm) metals constructed from traditional (Tt), digitally impressed tooth (Dt), digitally impressed model (Dm), and digitally milled wax (Dw) techniques, veneered with porcelain by layering (Pl) or press-on (Pp) method at as-cast (As), degassing (De), opaquing (Op), body contouring (Co), and glazing (Gl) stage at gingival (G), gingiva-axial (Ga), axial (A), axio-occlusal (Ao), and occlusal (O) location of the buccal (Bu), lingual (Li), mesial (Me), and distal (Di) sites

Metal	Veneer	Stage	Site																	
			BuG	BuGa	BuA	BuAo	Obl	LiAo	LiA	LiGa	LiG	MeG	MeGa	MeA	MeAo	Odl	DiAo	DiA	DiGa	DiG
CmTt	Pl	As	56.9	69	75	108.4	116.9	111	74.6	69.8	59.6	58.2	72.8	73.9	104.5	116.5	103.8	75.2	73.5	60.7
CmTt	Pl	De	71.1	82.9	86	127.9	137.2	130.2	85.3	84.3	72.5	73.8	87.2	83.2	124.9	137.2	124.3	85.8	88.3	75.8
CmTt	Pl	Op	72.9	85.2	88	130.3	140.4	132.7	87.2	86.2	74.5	75.8	88.7	84.8	126.7	140.1	126.6	87.7	90.2	77.9
CmTt	Pl	Co	77.3	90.1	91.6	135	145.6	136.9	90.4	89.9	78.8	79.8	92.7	88.2	131.5	145.6	131.2	90.8	93.92	81.8
CmTt	Pl	Gl	78.1	91.2	93	136.8	147.8	138.8	91.8	91.1	79.9	81.1	93.9	89.7	133.1	146.8	132.6	92.7	95.4	82.9
CmTt	Pp	As	58.7	69.1	74.7	110.2	117.4	110.5	74.7	70.9	59.3	61.3	69.8	77.3	109.4	117.5	109.2	75.9	70.9	60.1
CmTt	Pp	De	71.9	83.2	85.5	130.7	140	131.2	85.9	84.1	72.25	74.4	83.1	88.2	129.4	140.2	128.6	87	84.2	72.8
CmTt	Pp	Op	73.8	84.8	86.7	133.6	143.2	134.2	87.8	86	74.1	75.8	85.2	89.7	132.6	143.7	130.9	88.8	86.4	74.5
CmTt	Pp	Co	71.3	83.2	85.6	131.6	140.5	131.9	86.2	85.1	71.6	72.8	83.2	88.1	130.5	141.1	128.4	87.4	85.4	72.3
CmTt	Pp	Gl	73.5	85.3	87.7	134.6	144	134.8	87.8	87	73.9	74.9	85.1	90.1	133.7	144.4	131.2	89.3	87.6	74.5
CmDw	Pl	As	61.6	85.8	78.4	126.7	134.2	126.6	78.8	87.2	61.6	62.8	88.7	81.5	123.8	134.3	123.8	84.3	91.7	65.2
CmDw	Pl	De	74.8	98.3	88.8	147.3	155.2	146.2	90.5	100.2	74.5	75.5	101	91.6	144.2	155.3	143.8	94.5	103	78.2
CmDw	Pl	Op	76.9	100.8	90.9	150.6	158.3	149.2	92.2	102.2	76.7	77.4	103.2	93.4	147.3	158.6	146.4	96.2	105.3	80.5
CmDw	Pl	Co	80.9	106.1	95.7	156.6	164.6	156.2	96.1	106.3	80.6	81.7	108.5	97.4	154.2	164.6	152	99.9	109.3	84.2
CmDw	Pl	Gl	82.2	106.8	96.8	158.1	166.8	156.9	97.2	107.3	81.8	83.2	109.9	98.5	155.4	166.8	153.4	101.4	110.7	85.1
CmDw	Pp	As	64.2	85.8	78.2	126.2	130.8	128.6	79.5	87.2	63.3	67.8	88.9	79.5	121.7	131.1	121.2	79.7	89.8	67.2
CmDw	Pp	De	77.1	98.5	87.8	146.6	150.2	149.9	88.9	99.8	75	80	102	89.4	142.5	151.8	141.1	90.1	102.3	79.3
CmDw	Pp	Op	78.8	100.6	89.7	149.2	153.6	152.6	90.5	101.8	76.9	82	104.6	91.3	145.3	155.2	143.3	91.8	104.2	81.2
CmDw	Pp	Co	76.5	99.3	88.1	146.3	150.2	150.1	89.1	99.8	74.3	79.7	103.2	89.9	142.8	151.8	141.2	90.6	102.6	79.2
CmDw	Pp	Gl	79	101.8	89.7	149.3	153.3	150	90.9	102	76.8	82.5	105.2	91.5	145.2	155.9	143.8	92.5	104.2	81.2
SmDt	Pl	As	36.5	59.2	60.8	112.2	119.8	114.4	69.3	63.6	40.7	40.1	64	71.4	100.1	120.7	100.2	72.2	62.9	40
SmDt	Pl	De	47.9	71.7	69.4	131.3	139.1	133.5	77.9	75.6	49.2	48.9	77.1	80.3	119.2	139.5	120.2	83	76.2	48.4
SmDt	Pl	Op	49.4	73.9	71.8	134.1	142.3	135.8	80	77.8	51.1	50.6	79.2	82.3	121.8	142.5	122.8	84.8	78.3	49.9
SmDt	Pl	Co	53.9	78.5	76.2	138.9	148.5	141.3	84.2	82.2	54.9	54.7	83.8	86.6	127	149	128	88.6	82.2	53.5
SmDt	Pl	Gl	55.1	79.8	77.7	140.9	150.0	142.8	85.8	83.6	56	55.9	85.2	88	128.6	150.5	129.6	90.2	83.7	54.8
SmDt	Pp	As	40.1	62.9	61.3	112.2	119.5	112.4	63.8	64	40.2	40.6	66.2	67.8	101.1	119.3	102.6	69.5	66.4	41.7
SmDt	Pp	De	48.4	74.8	71.2	129.5	138.1	130.2	73.4	76.1	48.4	49.9	77.5	78	116.5	137.9	119.3	80.3	79.1	50.8
SmDt	Pp	Op	50.4	76.8	77.3	132.3	140.9	133.1	75.5	78.5	50.3	51.9	79.7	79.9	119.2	140.6	122	81.8	81.1	52.5
SmDt	Pp	Co	40.8	74.8	71.1	129.5	137.2	119.6	79.7	78.3	50.4	48.2	75.2	73.8	130.6	136.9	116.6	77.6	77.3	49.9
SmDt	Pp	Gl	50.6	77.3	73.5	132.2	140.6	133.4	75.9	78	50.9	52.3	80	79.5	120.1	140.3	122.6	82.2	81.2	53.1
SmDm	Pl	As	38.9	63.2	63.4	118.2	123.8	119.5	62.3	64.6	40.8	42.9	65.3	66.7	108	122.6	108.8	67.2	64.4	41.5
SmDm	Pl	De	47.3	75.2	73.7	135.2	144.1	135.7	72.7	76.6	49	51.4	77.1	76.5	124.5	143.8	127.1	78.1	76.3	50.1
SmDm	Pl	Op	49	76.8	75.7	138.1	147.3	138.7	74.4	78.2	50.7	53.2	78.8	78.9	127.5	147.1	130	79.9	78.1	51.3
SmDm	Pl	Co	53.3	80.5	79.9	142.7	152.9	143.6	78.3	81.8	54.3	57.2	82.8	82.5	131.8	152.4	134.2	83.5	81.9	55.2
SmDm	Pl	Gl	54.9	81.7	81.4	145.1	154.8	145.7	79.6	83.3	55.8	58.6	84.4	84.2	134	154.3	135.8	84.9	83.6	56.8
SmDm	Pp	As	39.8	67.8	65.4	118.2	124.8	120	64.4	66.1	40.5	40	66.6	67.7	108.6	124.2	110.5	65.2	53.8	38.8
SmDm	Pp	De	47.7	80.2	74.8	137.7	144.2	139	74.5	77.2	48.8	47.7	77.1	77.5	128	143.9	129.5	75	75.2	46.8
SmDm	Pp	Op	49	82.1	76.6	140.2	147.2	141.7	76.2	78.9	50.2	49.3	78.8	79.8	130	146.9	132	77.1	77.2	48.6
SmDm	Pp	Co	46.6	79.6	73.2	137.3	143.5	139.1	74.4	76.9	47.6	46.9	76.8	78	127.3	143.4	128.8	75.2	75	46.1
SmDm	Pp	Gl	49.1	81.8	75.2	140.3	146.3	142.2	76.4	79.1	50.1	49.2	79.7	80.4	130.8	146.7	131.6	77.4	77.8	48.8

**Table 2:** Analysis of variance (ANOVA) of internal accuracy at different sites of ceramic veneer metal substructures constructed from different techniques with different veneering methods upon various stages of fabrication

Source	SS	df	MS	*F*	*p*
Corrected model	8690094.324	719	12086.362	182.755	0.001
Intercept	78906147.426	1	78906147.426	1193120.795	0.001
Substructure	566193.110	3	188731.037	2853.756	0.001
Stage	410275.035	4	102568.759	1550.917	0.001
Ceramic	12376.884	1	12376.884	187.148	0.001
Site	7464066.605	17	439062.741	6638.962	0.001
Substructure * Ceramic	1157.590	3	385.863	5.835	0.001
Substructure * Site	169578.567	51	3325.070	50.278	0.001
Substructure * Stage	1795.834	12	149.653	2.263	0.007
Ceramic * Site	2010.522	17	118.266	1.788	0.024
Ceramic * Stage	21168.211	4	5292.053	80.020	0.001
Site * Stage	28827.965	68	423.941	6.410	1.000
Substructure * Ceramic * Site	9974.445	51	195.577	2.957	0.001
Substructure * Ceramic * Stage	114.923	12	9.577	.145	1.000
Substructure * Site * Stage	1204.666	204	5.905	.089	1.000
Ceramic * Site * Stage	642.664	68	9.451	.143	1.000
Substructure * Ceramic * Site * Stage	707.302	204	3.467	.052	1.000
Error	523783.250	7920	66.134		
Total	88120025.000	8640			
Corrected total	9213877.574	8639			

Abbreviations: df, degree of freedom; *F* , F-ratio; MS, mean square; SS, sum of squares.

**Fig. 4:**
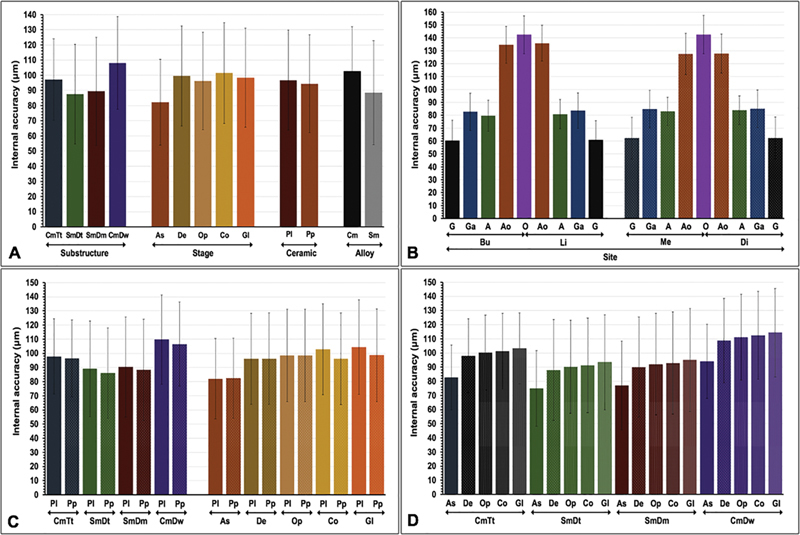
Internal accuracy as a function of ( **A** ) substructure fabrication techniques [cast metal constructed from the traditional impressed tooth (CmTr), sintered metal constructed from the traditional impressed model (SmDt), sintered metal constructed from the digitally impressed model (SmDm), and cast metal constructed from digitally impressed wax (CmDw)], stages of fabrication (as-cast: As; degassing: Ds; opaquing: Op; body contouring: Co; glazing: Gl), ceramic veneering method (layering: Pl; Press-on: Pp), alloy types (cast metal; Cm, sinter metal; Sm), ( **B** ) sites [gingival (G), gingiva-axial (Ga), axial (A), axio-occlusal (Ao), and occlusal (O) location of the buccal (Bu), lingual (Li), mesial (Me), and distal (Di) surface], ( **C** ) interaction of substructure fabrication techniques with ceramic veneering methods, interaction of stages and ceramic veneering methods, and ( **D** ) interaction of substructure fabrication techniques and stages of fabrication.

Post hoc Bonferroni multiple comparisons showed significant differences in the internal accuracy of restorations upon different factors tested ( Tables 3 and 4 ). The internal discrepancy of ceramometal restorations as a factor of the substructure fabrication techniques indicated that the CmDw (108.15 ± 30.6 µm) exhibited a significantly greater internal gap than that constructed from the CmTt (97.11 ± 26.86 µm), SmDm (89.42 ± 35.57 µm), and SmDt (87.58 ± 32.9 µm), respectively ( *p* < 0.05) ( Fig. 4A and Table 3A ). The internal discrepancy of ceramometal restorations as a factor of stages of fabrication revealed a significant difference in the internal gap among As (82.22 ± 28.34 µm), De (99.5 ± 33.01 µm), Op (96.2 ± 32.17 µm), Co (101.51 ± 33.23 µm), and Gl (98.4 ± 32.64 µm) stages ( *p* < 0.05) ( Fig. 4A and Table 3B ). The internal discrepancy of ceramometal restorations as a factor of ceramic veneering methods demonstrated a significantly larger internal gap upon veneering with conventional layering (Pl) (96.76 ± 32.99 µm) compared to the pressed-on veneering (Pp) (94.37 ± 32.28 µm) methods ( *p* < 0.05) ( Fig. 4A and Table 3C ). The statistics also indicated significantly better internal accuracy upon the metal substructures fabricated from sintered metal (Sm, 88.50 ± 34.27 µm) compared to cast metal (Cm, 102.63 ± 29.31 µm) ( *p* < 0.05) ( Fig. 4A and Table 3D ). The internal discrepancy of ceramometal restorations as a factor of sites of restoration demonstrated a significantly larger internal gap from the gingival (G), axial (A), gingivo-axial (Ga), axio-occlusal (Ao), and occlusal (O) locations, respectively, for both buccal (Bu), lingual (Li), mesial (me), and distal (Di) sites ( *p* < 0.05). Nevertheless, no significant difference in internal discrepancy of restoration was found between BuG-LiG, MeG-DiG, BuA-LiA, BuGa-LiGa-MeA-DiA, MeGa-DiGa, MeAo-DiAo, BuAo-LiAo, and O sites of Bu-Li and Me-Di directions ( *p* > 0.05) ( Fig. 4B and Table 3E ). Post hoc Bonferroni multiple comparisons signified significant differences in internal accuracy upon the different combinations of substructure fabrication techniques and methods of ceramic veneering ( *p* < 0.05), except between CmDwPl–CmDwPp, CmTtPl–CmTtPp, SmDmPl–SmDmPp–SmDtPl, and SmDtPl–SmDtPp–SmDwPp groups ( *p* > 0.05) ( Fig. 4C and Table 4A ). Bonferroni multiple comparisons signified a significant difference in internal accuracy upon the different combination of stages of restoration fabrication and methods of ceramic veneering ( *p* < 0.05), except between AsPl–AsPp, DePl–DePp–OpPl–OpPp–CoPp–GlPp, and CoPl–GlPl groups ( *p* > 0.05) ( Fig. 4C and Table 4B ). Bonferroni multiple comparisons signified significant difference in internal accuracy between the combination of substructure fabrication techniques and stages of restoration fabrication ( *p* < 0.05), except between CmTtAs–SmDtAs–SmDmAs groups, CmTtDe–CmTtOp–CmTtCo–CmTtGl groups, SmDtDe–SmDtOp–SmDtCo–SmDtGl–SmDmDe–SmDmOp–SmDmCo–SmDmGl–CmDwAs groups, and CmDwDe–CmDwOp–CmDwCo–CmDwGl groups ( *p* > 0.05) ( Fig. 4D and Table 4C ).

**Table 3:** Post hoc Bonferroni multiple comparisons of internal accuracy of ceramic veneer alloy as a factor of (A) substructures constructed from (cast: Cm; sintered: Sm) metal upon different techniques (traditional: Tt; digitally impressed tooth: Dt; digitally impressed model: Dm; and digitally milled wax: Dw), (B) stages of fabrication (as-cast: As; degass: De; opaquing: Op; body contouring: Co; glazing: Gl), (C) ceramic veneering methods (layering: Pl; Press-on: Pp), (D) alloys (cast: Cm; sintered: Sm), and (E) sites (at gingival: G; gingiva-axial: Ga; axial: A; axio-occlusal: Ao; and occlusal: O locations of the buccal: Bu; lingual: Li; mesial: Me; distal: Di surface)

**(A) As a factor of substructures**										**(B) As a factor of fabrication stages**						**(C) As a factor of ceramic**			
**Substructure**		**CmTt**		**SmDt**		**SmDm**		**CmDw**		**Stage**	**As**	**De**	**Op**	**Co**	**Gl**	**Ceramic**		**Pl**	**Pp**
CmTt		1		0.001		0.001		0.001		As	1	0.001	0.001	0.001	.001	Pl		1	0.001
SmDt				1		0.001		0.001		De		1	0.001	0.001	.001	Pp			1
SmDm						1		0.001		Op			1	0.001	.001	**(D) As a factor of alloys**			
CmDw								1		Co				1	.001	Alloy		Cm	Sm
										Gl					1	Cm		1	0.001
																Sm			1
**(E) As a factor of sites**																			
**Site**		**Bu**				**O**	**Li**				**Me**				**O**	**Di**			
		**G**	**Ga**	**A**	**Ao**	**O**	**Ao**	**A**	**Ga**	**G**	**G**	**Ga**	**A**	**Ao**	**O**	**Ao**	**A**	**Ga**	**G**
Bu	G	1	0.001	0.001	0.001	0.001	0.001	0.001	0.001	1	0.030	0.001	0.001	0.001	0.001	0.001	0.001	0.001	0.022
	Ga		1	0.001	0.001	0.001	0.001	0.190	1	.001	0.001	0.004	1	0.001	0.001	0.001	1	0.001	0.001
	A			1	0.001	0.001	0.001	1	0.001	0.001	0.001	0.001	0.001	0.001	0.001	0.001	0.001	0.001	0.001
	Ao				1	0.001	1	0.001	0.001	0.001	0.001	0.001	0.001	0.001	0.001	0.001	0.001	0.001	0.001
O	O					1	0.001	0.001	0.001	0.001	0.001	0.001	0.001	0.001	1	0.001	0.001	0.001	0.001
Li	Ao						1	0.001	0.001	0.001	0.001	0.001	0.001	0.001	0.001	0.001	0.001	0.001	0.001
	A							1	0.001	0.001	0.001	0.001	0.013	0.001	0.001	0.001	0.001	0.001	0.001
	Ga								1	0.001	0.001	1	1	0.001	0.001	0.001	1	0.048	0.001
	G									1	0.014	0.001	0.001	0.001	0.001	0.001	0.001	0.001	0.913
Me	G										1	0.001	0.001	0.001	0.001	0.001	0.001	0.001	1
	Ga											1	0.071	0.001	0.001	0.001	0.022	1	0.001
	A												1	0.001	0.001	0.001	1	0.017	0.001
	Ao													1	0.001	1	0.001	0.001	0.001
O	O														1	0.001	0.001	0.001	0.001
Di	Ao															1	0.001	0.001	0.001
	A																1	0.016	0.001
	Ga																	1	0.001
	G																		1

**Table 4:** Post hoc Bonferroni multiple comparisons of internal accuracy of ceramic veneer alloy as a function of (A) the interaction of substructures (cast: Cm; sintered: Sm) metal constructed from different techniques (traditional: Tt; digitally impressed tooth: Dt; digitally impressed model: Dm; and digitally milled wax: Dw) and ceramic veneering methods (layering: Pl; Press-on: Pp), (B) the interaction of stages of fabrication (as-cast: As; degass: De; opaquing: Op; body contouring: Co; glazing: Gl) and ceramic veneering methods, and (C) the interaction of substructures constructed from different techniques and stages of fabrication

**(A) As an interaction of substructures and ceramic**										**(B) As an interaction of stage of fabrication and ceramic**											
Sub*Por		**CmTt**		**SmDt**		**SmDm**		**CmDw**		Stage*Por		**As**		**De**		**Op**		**Co**		**Gl**	
		**Pl**	**Pp**	**Pl**	**Pp**	**Pl**	**Pp**	**Pl**	**Pp**			**Pl**	**Pp**	**Pl**	**Pp**	**Pl**	**Pp**	**Pl**	**Pp**	**Pl**	**Pp**
CmTt	Pl	1	1	0.001	0.001	0.001	0.001	0.001	0.001	As	Pl	1	1	0.001	0.001	0.001	0.001	0.001	0.001	0.001	0.001
	Pp		1	0.001	0.001	0.001	0.001	0.001	0.001		Pp		1	0.001	0.001	0.001	0.001	0.001	0.001	0.001	0.001
SmDt	Pl			1	0.842	1	1	0.001	0.001	De	Pl			1	1	1	1	0.001	1	0.001	1
	Pp				1	0.043	1	0.001	0.001		Pp				1	1	1	0.010	1	0.001	1
SmDm	Pl					1	1	0.001	0.001	Op	Pl					1	1	0.016	1	0.005	1
	Pp						1	0.001	0.001		Pp						1	0.018	1	0.005	1
CmDw	Pl							1	0.052	Co	Pl							1	0.001	1	0.028
	Pp								1		Pp								1	0.001	1
										Gl	Pl									1	0.010
											Pp										1
**(C) As an interaction of substructure and stage of fabrication**																					
Sub*Stage		**CmTt**					**SmDt**					**SmDm**					**CmDw**				
		**As**	**De**	**Op**	**Co**	**Gl**	**As**	**De**	**Op**	**Co**	**Gl**	**As**	**De**	**Op**	**Co**	**Gl**	**As**	**De**	**Op**	**Co**	**Gl**
CmTt	As	1	0.001	0.001	0.001	0.001	0.147	1	0.066	0.008	0.001	1	0.105	0.002	1	0.001	0.001	0.001	0.001	0.001	0.001
	De		1	1	1	1	0.001	0.001	0.037	0.024	1	0.001	0.023	0.035	1	1	1	0.001	0.001	0.001	0.001
	Op			1	1	1	0.001	0.001	0.001	0.004	0.023	0.001	0.001	0.022	0.100	0.015	0.003	0.009	0.001	0.001	0.001
	Co				1	1	0.001	0.001	0.001	0.001	0.032	0.001	0.001	0.002	0.012	0.028	0.114	0.017	0.001	0.001	0.001
	Gl					1	0.001	0.001	0.001	0.001	0.001	0.001	0.001	0.001	0.001	0.019	0.003	1	0.038	0.002	0.001
SmDt	As						1	0.001	0.001	0.001	0.001	1	0.001	0.001	0.001	0.001	0.001	0.001	0.001	0.001	0.001
	De							1	1	1	1	0.001	1	1	1	0.153	0.713	0.001	0.001	0.001	0.001
	Op								1	1	1	0.001	1	1	1	1	1	0.001	0.001	0.001	0.001
	Co									1	1	0.001	1	1	1	1	1	0.001	0.001	0.001	0.001
	Gl										1	0.001	1	1	1	1	1	0.001	0.001	0.001	0.001
SmDm	As											1	0.001	0.001	0.001	0.001	0.001	0.001	0.001	0.001	0.001
	De												1	1	1	1	1	0.001	0.001	0.001	0.001
	Op													1	1	1	1	0.001	0.001	0.001	0.001
	Co														1	1	1	0.001	0.001	0.001	0.001
	Gl															1	1	0.001	0.001	0.001	0.001
CmDw	As																1	0.001	0.001	0.001	0.001
	De																	1	1	1	1
	Op																		1	1	1
	Co																			1	1
	Gl																				1

## Discussion

Although carefully prepared tooth abutments as well as a well-controlled process of restoration construction, imprecision remains between the restorations and the prepared abutments, which predisposes to caries and periodontal disease. 13 The more precisely the fit of the restoration adapts to the prepared tooth, the smaller the internal gap displays, the slighter the cement film is bared to oral fluid, as well as the better the retentive and the resistance to dislodgement of the restoration. This study partially rejected the null hypothesis as there were significant differences in internal accuracy of ceramometal crown upon metal substructure fabrication techniques, stages of restoration fabrication, methods of ceramic veneering, sites of restoration, and two factors interaction except for sites and stages of restoration fabrication. However, no significant differences in the internal accuracy of ceramometal crown upon three- and four-factor interaction were found, except only the interaction of substructure fabrication technique, ceramic veneering methods, and site of restoration. Hence, the alternative hypothesis was partially confirmed. However, the internal gap for all the studied groups was less than 200 μm at the occlusal region and less than 100 μm at other regions, which is considerably accepted for internal misfit limit. 1 25 26

The metal substructures fabricated either from CmTt or CmDw techniques exhibited less internal accuracy than those fabricated either from SmDt or SmDm techniques, which were consistent with other studies. 1 3 4 20 This could be attributed to the conventional lost wax cast technique, which is a complex, sensitive, nonreproducible method, and requires high skill of dental technician to achieve a precise fit of restoration. The internal discrepancy of metal substructures constructed from the CmTt technique is primarily associated with the dimensional accuracy of the conventional processes in the construction of the restoration including the traditional impression technique of the prepared tooth (Tt), dimensional accuracy of the stone model, and investing material together with the solidification shrinkage volume of metal upon casting that directly influenced the restorations fit. 29 The internal discrepancy of the CmDw technique demonstrated a higher internal discrepancy than the CmTt technique, which is probably related to the milled wax substructure that additionally increases error accumulation in this process. 22 This was supported by other studies that reported the internal accuracy of the wax coping produced by hand carving was more precise than the wax coping produced by digitally milled wax. 5 8 The study indicated that metal substructure produced from sintered metal (Sm) provided better internal accuracy than cast metal (Cm). This study was in agreement with other previous studies. 4 7 9 The metal substructures constructed from the digital impression procedure either from the intraoral scanner for making a digital impression of the prepared tooth or the laboratory scanner for performing a digital impression of the stone die of the stone model were both designed for the copings using a three-dimensional software program and then milled the presintered metal blank with a CAM milling machine. These techniques do not involve the process of lost wax and for that reason, the stable dimensions seem to be achieved. Although fully sintered metal substructures must involve the shrinkage of powder metal upon sintering, the compensation was planned during CAD-designed substructures, which is considerably negligible. 6 The digital impression process normally creates somewhat rounded borders related to the resolution for each scanner, which probably causes an early contact of restoration at the axial-occlusal edges and causes a larger internal gap. The process of digital impression-taking usually makes the overshooter peak around the edges of the target and causes a higher internal inaccuracy. 10 This occurrence was expressed in every single CAD-CAM that involves digital impressions. 6 The cloud points gained from the digitally scanned process were converted into a continuously smooth surface depending on the proficiency of the designed software. This procedure can also steer to some impreciseness. However, the process of milling presintered metal blank is quite soft, seems easy, is not prone to establish internal cracking, and has less stress accumulation on the presintered substructure. 1 7 20 The internal accuracy of sintered metal substructures was comparable with either the presintered metal constructed from the STL file derived from digitally impressed tooth or digitally impressed model. This is probably related to the preciseness of the prepared tooth and the stone die was comparable. The result was consistence with other studies that found no significant difference between the digital impression of the prepared tooth and the prepared stone die. 30 Furthermore, the study signified that the restoration constructed from sintered metal revealed superior internal fit than those constructed from cast metal as supported by other studies. 4 7 9

Concerning the stages of restoration fabrication, the internal accuracy of the restoration was affected by the sequential stages of fabrication. Before veneering with ceramic, the Sm copings revealed superior internal preciseness than the Cm copings as supported by other studies. 1 2 A significant increasing internal gap of restorations upon as-cast to degassing, opaquing, contouring, and glazing process was evidenced, which corresponded with other studies. 1 11 18 21 The greatest increasing internal gap was found after the degassing process. Metal substructures exposed to extremely high temperatures during the porcelain firing stage possibly produce dimensional distortion and finally decrease the preciseness of the restoration. The result of this study corresponded with other studies that stated the greatest distortion of ceramic veneered metal occurs during the degassing stage. 11 18 21 This study used nonnoble metal alloy for fabricated metal substructure because of low cost, biocompatibility, resistance to corrosion, and stable in biological environments. However, the inherited disadvantage of nonnoble metal alloy is the thick oxide layer formation on the surface upon the degassing process. The degassing process took place at an elevated temperature and could cause the grain growth of the deformed crystals and was postulated to cause greater internal discrepancy upon degassing due to the composition of metal substructure as confirmed by other studies. 17 21 The increase in internal gap after sequential ceramic sintering generally influenced by numerous factors, for example, the ceramic firing shrinkage, the coefficient of thermal expansion (CTE) of metal (Cm = 14.5 × 10 ^−6^ K ^−1^ , Sm = 14.5 × 10 ^−6^ K ^−1^ ), and ceramic (Pl = 12.9 ± 0.5 × 10 ^−6^ K ^−1^ , Pp = 13.2 ± 0.5 × 10 ^−6^ K ^−1^ ), and the residual stresses generated from multiple firing processes. The CTE differences of Pl to both alloys were slightly larger than the CTE differences of Pp to those alloys. This probably induced higher residual stress on the layering system than the press-on system, and caused a larger internal gap for the layering groups compared to the pressed-on groups, as supported by other studies. 1 5 However, this study indicated that internal discrepancies occurred after ceramic veneering, ceramic firing shrinkage as a causative factor in the internal misfit, were not primary factors in distortion as in agreement with other studies. 1 15 22

The ceramic veneering methods could generate different internal discrepancies of metal-ceramic restorations, which exhibited less internal distortion upon veneering with pressable ceramics. The internal accuracy of restoration veneered with pressed-on ceramic (Pp) was better than restoration veneered with conventional layering ceramic (Pl), which was supported by other studies. 12 22 23 This might be the effect of the number of ceramic firing cycles. The conventional ceramic veneering technique normally requires more firing cycles and higher skillfulness of dental technicians than the pressed-on techniques during the dentine porcelain contouring process. The press-on ceramic veneering technique requires full contour wax-up of the restoration on the substructure and then replaces it with pressed ceramic. This technique also eliminates technical errors from the porcelain firing process and multiple firings in conventional ceramic layering, thus reducing accumulated internal discrepancies. 23 Increased internal gap was reported from contamination of ceramic at the intaglio surface of metal substructure upon ceramic layering, while this event never occurred in the press-on technique. 22

A larger internal gap was exhibited at the occlusal region than at the axio-occlusal, axial, gingiva-axial, and gingival locations, respectively. This is probably associated with the configuration of the restoration shrinkage through the processes of fabrication that are exhibited in three dimensions. The characteristics of firing shrinkage occur toward the center of the restoration and cause inaccuracy on the occlusal and proximal sites more than on the other sites as other reports. 19 24 There are many techniques for evaluating internal accuracy of restorations such as direct viewing, cement thickness measurement, cone-beam computed tomography, and silicone replica technique. 28 This study uses the silicone replica technique because it provides several advantages of the measuring process without destroying the specimen, repeating the measurement, high reliability, and precision. 14 28 Nevertheless, the color of the silicone replica should be extremely different from the color of the pick-up silicone used for sandwiching replica to mitigate error during evaluation of the internal gap. Since this study used the prepared premolar size metal tooth abutment that has the preparation conforming with the geometry of the tooth, thus the locations of the internal accuracy were assigned at 18 sites on the bucco-lingual and mesio-distal direction to eliminate the confounding effect from sectioning silicone replica upon other sites. Thus, it is suitable for the evaluation of internal accuracy at different stages of fabrication in this study.

Since ceramic veneer sintered metal is comparatively new and rapidly utilized in clinical practice, there are several techniques to derive for final restorations. The information on the accuracy of restoration in this experiment founded on the systematized circumstances concerning the study design, metal substructure fabrication techniques, ceramic veneering methods, method of evaluation, and experimental implementation providing realistically important scientific value for dentists in decision-making for dental reconstruction using digital approached ceramic veneer metal restoration in their dental practices. Nevertheless, upon the study's limitations, sintered metal substructures either fabricated from digitally impressed tooth or digitally impressed stone model, whether ceramic veneering by layering or press-on methods, provided better internal accuracy than cast metal substructures either fabricated from traditional lost wax or digitally milled wax, even if ceramic veneering by layering or press-on technique. However, the operator and the equipment calibration need to be considered, which could influence the results. Nevertheless, all techniques described in the study satisfied precise internal fit and were clinically acceptable for the fabrication of ceramic veneer metal restorations for oral reconstruction. 14 25 26 Still, the effects of different types of dental cement and the potential impact of internal accuracy of ceramometal restoration on the long-term clinical use need to be further investigated.

## Conclusion

This study confirmed that the internal accuracy of ceramometal restorations was significantly affected by the different metal substructure fabrication techniques, ceramic veneering methods, sequential stages of restoration fabrications, and sites of restorations. Sintered metal substructures either fabricated from digitally impressed tooth (SmDt) or digitally impressed stone models (SmDw) achieved better internal accuracy than cast metal substructures either fabricated from traditional lost wax (CmTt) or digitally milled wax (CmDw). Ceramic press-on metal substructure generated better internal accuracy than the conventional ceramic layering method. A continued increase in internal inaccuracies of ceramic veneer metal through the sequential restorative fabrication processes was addressed, with the greatest increasing internal inaccuracies occurring during the degassing process and sequential ceramic sintering. Higher internal inaccuracies were exhibited more on the occlusal and axio-occlusal sites than on the other sites. Nevertheless, the ceramic veneered metal restoration fabricated in this study has shown clinically acceptable internal accuracy. The study suggests fabricating ceramometal restoration with a sintered metal substructure and veneered with a ceramic press-on technique to derive suitable internal accuracy for restorative reconstruction.
